# The benefits and hazards of psychodrama in the management of mental illness: qualitative study linked to nidotherapy

**DOI:** 10.1192/bjb.2024.57

**Published:** 2025-06

**Authors:** Zenab Ahmed, Peter Tyrer

**Affiliations:** 1 University of Lincoln, UK; 2 Imperial College London, UK; 3 Nottingham Trent University, UK

**Keywords:** Qualitative research, arts psychiatry, psychological treatments, psychosocial interventions, social functioning

## Abstract

Drama therapy is a popular form of management in mental illness, as it reaches out beyond many other therapies. Few studies have examined both the advantages and disadvantages of this medium. This qualitative study examines both, and finds gains and hazards.

Psychodrama and drama therapy, ever since Moreno^1^ introduced the concepts, have offered opportunities to address possible solutions to personal and interpersonal problems through the actions of drama rather than words. They have also been adopted as aspects of the environmental treatment known as nidotherapy, the management of symptoms and behaviour through personal planned change in the environment.^2^ Nidotherapy is carried out mainly with individuals choosing an environmental pathway in discussion with a nidotherapist. It has shown positive but not unequivocal value in case reports,^3,4^ two randomised controlled trials^5,6^ and one qualitative study.^7^

However, in classical nidotherapy, the patient chooses an environmental option that has already been desired or is addressed through examination of competing elements.^2^ The option of acting and psychodrama is rarely chosen as an option and comes up in discussion, especially in those with limited self-confidence and uncertainty about their place in society and their occupational status. This dilemma also applies in clinical practice. Whereas psychodrama is focused on emotional self-understanding, drama therapy offers an escape from the drudgeries of life to illustrate that self-worth can be gained by transforming into somebody else and all the excitement that this can bring,^8^ and so it achieves self-understanding in a different way. Most patients cannot see this option in advance; it has to be introduced and explained. However, an enthusiastic drama therapist may persuade a patient inappropriately to take part in dramatic action, only for it to backfire. We therefore thought it useful to evaluate the factors that might help choice in drama therapy by a detailed examination of its impact on patients who had received drama as individual therapy in the course of tackling their mental health problems.

In nidotherapy, patients with any form of mental illness who feel that environmental change is appropriate in their lives are assessed by investigation of their environmental wishes,^2^ their personality strengths and weaknesses,^9^ and their degree of motivation. The evidence for the benefit of nidotherapy has been explored in several studies. It is an indirect social prescribing intervention that accommodates needs and concerns relating to the patient's personal, social and physical environment.^10^ There are four aspects of the nidotherapy framework: person–environment understanding, environmental analysis, creation of a new environmental pathway (nidopathway) and monitoring of the pathway. Drama therapy is one of the components of the creation of a nidotherapy but can often precede it during environmental analysis. It can also be part of the monitoring of progress.^2^

In the assessment of nidotherapy a set of options are made available to the patient as the component of environmental analysis.^11^ Many already have very clear ideas and aims, but the wish to take part in dramatic productions is not normally on the list. However, when it is clear that activities are wished that promote social inclusion^12^ and escape from imposed restrictions in current settings,^4^ then the option of drama can be attractive. It was first used when introduced to in-patient care in a whole-hospital approach^13^ and has been a constant element since (www.nidotherapy.co.uk). It differs from other components of nidotherapy as it is not normally put forward as an environmental wish. It therefore has the potential of being seen as an imposed change that might have negative consequences. This was a relevant aspect of the present study.

In our study, we evaluated the impact of drama therapy on patients who had been involved in psychodrama as part of their nidotherapy management at some point in the recent past.

## Method

### Patients

Five male patients aged between 25 and 57 years, under the care of a registered mental health charity, NIDUS-UK, were involved in the study. For all five patients, it had been decided that drama therapy could be part of their nidotherapy programme. Three of the patients had taken part in drama productions previously, but during the study all had been involved in taking part in or rehearsing for two radio plays developed from an historical novel^14^ and a live musical play, *The Battle of Stoke Field.*^15^ All the patients gave consent for the study after receiving a participation information sheet.

Two of the patients had been unwell with significant depressive illness, one had a past history of psychosis linked to drug misuse but was now improved, the fourth had chronic social anxiety, and the last suffered from emotional instability and had alcohol dependence. Patients in NIDUS-UK are assessed using the ICD-11 diagnostic system; the five diagnoses were single-episode depressive disorder (6A70), recurrent depressive episode (6A71), social anxiety disorder (6B04), substance use psychosis due to cannabis (6C41.6) and moderate personality disorder with disinhibition (6D11.3).^16^

All the patients had been known to P.T. before the interviews, and this is relevant to their responses. Three had been involved in acting performances in other plays written by P.T. and carried out as part of nidotherapy (www.nidotherapy.co.uk).^17^ As a consequence, they had had time to reflect on the influence of drama in the progress of their mental illness and were not ingénues to the medium. The initial buzz of being involved in a dramatic performance in front of an audience had been tempered by later experience and allowed more considered responses to be made at interview. In each of the cases described, there was a joint purpose: to use drama therapy as a means of managing a complex mix of doubt, despair and feelings of futility; and also to determine whether improved self-esteem could follow.

### Gathering of data and interview procedure

Each of the participants was interviewed at least once by P.T. or Z.A. They were chosen as the only current patients in NIDUS-UK who were taking part in drama. All the data were analysed by Z.A. alone. The interviews were conducted by a combination of home visits and video calls (Zoom) and covered between 1 and 2 h. The interviews were semi-structured but were constructed around the main themes associated with both present and past experiences of drama. The interview procedure was flexible, allowing each participant to explore the research question in their own way and using their own terms. This avoided the handicaps of formal approaches to data collection that can become rigid and restricted.

### Thematic analysis

The various approaches to thematic analysis involve inductive and deductive approaches. In this research, we used an inductive approach, in which the data determined the themes of the results, and an open-ended framework to allow codes to reflect the content. Likewise, our interpretation of the data was coded on a semantic level for analysis of the explicit content of the data, followed by thematic analysis.^18^

No notes were taken during the interview, to promote active listening; afterwards, the researcher listened to the interview recordings multiple times to achieve familiarity with the content. The data were analysed with CAQDAS (computer-assisted qualitative data analysis software), and NVivo 13 was used to code data from the transcripts and convert them into themes and subthemes. While coding, the researcher tried to find the meaning of the key theme words through the participant's interpretation and experience.

### Research method

The chosen qualitative research methods mainly included interview transcripts. The interviews were carried out informally as ‘narrative occasions’^19^ to help participants to show their stories through conversation.

### Content of interviews

The interviews were organised in a semi-structured manner to understand patients’ personal experiences. The semi-structured interview style allowed for a more conversational flow with the participants, whereby they talked without any interruptions. This approach of discourse cultivates ‘reciprocal and empowering’ interactions with patients.^20^

The interviews were recorded using either a portable phone as specified or a camera and sent via a secure encrypted link. Interviews were transcribed verbatim, analysed via coding and then put into theme headings. During coding, the researcher determined the most economical way of summarising the key themes identified through the participant's interpretation and experience.^21^

### Ethical approval

Ethical approval was granted by the University of Nottingham Research Ethics Committee (reference number FMHS 91-1022).

## Results

The thematic analysis yielded five positive and four negative themes (Table 1).

**Table 1:** Main themes identified in qualitative analysis

Positive themes	Negative themes
Personal development	Devaluation
Better motivation	Vulnerability
Catharsis	Self-consciousness
Social cohesion	Negative emotion release
Insight	

### Positive themes

- Personal development. The novelty of dramatic performance was seen by three of the patients as helpful in their personal development. Comments such as ‘I got a sense of purpose’ and ‘became fascinated by the reaction’ when performing indicated the nature of this novelty. In one case, a patient aged 37, it had led to him becoming inspired to try to become a professional actor. ‘I just realised that everything else was unimportant compared with acting.’
- Motivation. One of the major consequences of chronicity in mental illness is loss of motivation. Most of the participants found that acting added inspiration to their lives and helped to put their own problems into ‘bite-size chunks’ that they could tackle in a systematic way. At times of particular adversity in their lives, the reminder of previous acting roles reinforced their resolve.
- Catharsis. Acting allows many emotions that are repressed to be give expression. ‘It is a very good way of releasing all my feelings,’ said one participant, and in the setting of a dramatic performance this can be not only allowed but actively encouraged in many roles. As one patient put it: ‘having the space to just to be me makes me feel safe and secure’.
- Social cohesion. One of the advantages of drama is the coming together of people from very different backgrounds in a setting where hierarchical structures are minimal. On participant described this as ‘part of a collective’ that was ‘greater than themselves’ and so felt reinforced by contact with others in the cast.
- Insight. Greater understanding of your own problems can often be gained in drama therapy. Placing the problems outside the realm of your own experience and tuning in to another character can be illuminating. As a participant put it, ‘I just think that playing those characters, there's always some light in there somewhere. So even though on the outside they might seem dark and cynical, there's always some goodness. So just because I was playing these, these darker characters didn't mean that there wasn't joy in them somewhere. You just have to find it.’

All five participants contributed to these themes.

### Negative themes

- Devaluation. The actors sometimes felt childish in doing something that the person would not do normally and imagined others would think they were acting in silly or stupid way. ‘I just felt it was not making enough demands on me; it was not intellectually stimulating enough’ was how one participant described it, and he was pleased that members of his family were not looking on.
- Vulnerability. This was a major concern with all the participants in the survey and is linked to the common notion of fear of failure. All actors are acutely aware of audience reaction, and if this is not positive there can be a severe rebound of emotion. Comments such as ‘when you put so much of yourself into the acting and it can be heart-breaking if the audience do not respond as you hope, and the consequence can be that you finish up being demoralised and deflated and want to abandon acting altogether’. This was particularly prominent in the patient with social anxiety.
- Self-consciousness. Anxious and nervous individuals are often put off by the public image of acting, and this leads to discouragement. In the words of one participant, ‘I felt very self-conscious that I was being looked at, and it seemed as though this sort of got bigger, like a snowball going down a hill, it got bigger and bigger and got to the point where I was so embarrassed I stopped and gave up.’
- Negative emotional release. Repressed anger is very common in mental illness, and although it can be useful to release it (see ‘Catharsis’ above), it can be overrepresented. As one participant put it, ‘if I am annoyed with people or generally feel pent up about something, I can overstate it and say or do things which I wouldn't do normally’.

Three of the five participants contributed to these themes.

## Discussion

This study had the disadvantage of having a small sample, all of whom were male, and so there may be gender distinctions that were not detected. There was partial compensation in that most of the participants were well versed in dramatic productions and so had an understanding of their value in advance through being involved in nidotherapy, often over a period of several years – a relevant period, as the effects of nidotherapy can be both delayed and long-lasting.^4,6^ It could therefore be concluded that they had a more rounded view of the subject than most when exposed to drama therapy.

There seems little doubt that drama therapy can have positive effects overall, especially improving personality strengths and self-esteem,^22^ but there are few published reports about the negative aspects of drama therapy. Cheung et al^23^ reported a sense of pride and the value of a safe space in dramatherapy but also commented on the major problem of hesitancy in many when invited to take part. They overcame this by using a remote online approach (the Co-active therapeutic Theatre model),^24^ which was linked to an independent recovery model using the patient's own narrative and self-perception.^25^

This may have had an influence on the reported disadvantages of dramatherapy. Most of the published work on dramatherapy describes uncontrolled studies by enthusiasts for the treatment – this is to be expected and is not surprising, and in such environments adverse effects tend to be downplayed and often not reported.^26^ On the other hand, the benefits tend to be well described, including reduced violence in forensic patients,^27^ reduction in self-stigma^28,29^ and exploration of trauma.^30^ Cheung et al^24^ found five positive themes associated with an online drama intervention that were very similar to the five themes found in our study – greater creativity, individual advocacy, improved self-confidence, better social connections and improved self-awareness – but did not report any negative impacts. The need for some reflection on potential negative effects needs to be taken into account both in practice and in future studies.

There is evidence of value of the use of drama therapy as an environmental option in nidotherapy, but the results of this study point to some negative implications that may be inhibiting. More care might be given to screening candidates before drama therapy is offered.

## Data Availability

Selected data from the study are available from Z.A.

## About the authors

**Zenab Ahmed** is a medical student at the University of Lincoln, UK. **Peter Tyrer** was a visiting lecturer at the University of Lincoln, UK, at the time of this project, and is an Emeritus Professor of Community Psychiatry at Imperial College London, UK and a visiting professor at Nottingham Trent University, UK.
